# Joint User Association and Deployment Optimization for Energy-Efficient Heterogeneous UAV-Enabled MEC Networks

**DOI:** 10.3390/e25091304

**Published:** 2023-09-07

**Authors:** Zihao Han, Ting Zhou, Tianheng Xu, Honglin Hu

**Affiliations:** 1 Shanghai Advanced Research Institute, Chinese Academy of Sciences, Shanghai 201210, China; hanzihao2019@sari.ac.cn (Z.H.); zhouting@shu.edu.cn (T.Z.); xuth@sari.ac.cn (T.X.); 2 University of Chinese Academy of Sciences, Beijing 100049, China; 3 School of Microelectronics, Shanghai University, Shanghai 200444, China

**Keywords:** UAV, MEC, UAV deployment, user association, energy-efficient, optimal transport theory, dragonfly algorithm

## Abstract

Unmanned aerial vehicles (UAVs) providing additional on-demand communication and computing services have become a promising technology. However, the limited energy supply of UAVs, which constrains their service duration, has emerged as an obstacle in UAV-enabled networks. In this context, a novel task offloading framework is proposed in UAV-enabled mobile edge computing (MEC) networks. Specifically, heterogeneous UAVs with different communication and computing capabilities are considered and the energy consumption of UAVs is minimized via jointly optimizing user association and UAV deployment. The optimal transport theory is introduced to analyze the user association sub-problem, and the UAV deployment for each sub-region is determined by a dragonfly algorithm (DA). Simulation results show that the energy consumption performance is significantly improved by the proposed algorithm.

## 1. Introduction

The emergence of intelligent applications, such as intelligent transportation systems, VR (virtual reality) and AR (augmented reality), has led to an increasing demand for on-demand communication and computing services beyond 5G/6G [1]. However, mobile devices still face challenges due to limited resources, such as battery life and computing power. This can be particularly difficult in emergency scenarios where infrastructure is lacking, making it difficult for mobile devices to be covered by terrestrial base stations and process computation-intensive and delay-sensitive applications [2]. To address these challenges, a new platform is needed that can provide high computation and communication resources, support massive connectivity, and ensure ultra-reliability and high throughput in remote areas or during disasters.

### 1.1. Related Works

To this end, Unmanned Aerial Vehicle (UAV)-aided Mobile Edge Computing (MEC) has obtained significant attention. This approach involves equipping UAVs with communication devices and computing servers to offer ground users ubiquitous and flexible services [3,4]. UAVs can adjust their location for specific purposes such as energy conservation and increased throughput. In addition, UAVs can also be less affected by fewer channel impairments due to their high altitude, which creates high possibility of line-of-sight (LoS) links with ground users and strengthens their coverage.

User association, as a widely used technique in wireless networks, has attracted a lot of research interest in UAV-aided wireless networks. It involves selecting users or groups of users to grant access to the available resources at a given time based on various criteria such as channel conditions, quality of service requirements, or fairness considerations. In [5], Qiu et al. studied a joint placement, resource allocation, and user association problem for UAV-aided wireless networks with constrained backhaul links. In [6], Zhu et al. proposed a multi-agent deep deterministic policy-gradient-based solution to optimize the flight trajectory, the association between the UAVs and user devices, and the task association of the user devices. In [7], Mozaffari et al. first introduced optimal transport theory to optimize cell association in UAV-enabled wireless networks, which yields improvements in terms of the average network delay. On this basis, ref. [8] extended the application of the optimal transport theory to the field of UAV-enabled MEC networks to minimize the total energy consumption.

The deployment locations of UAVs greatly affect system performance. In [9], Sun et al. proposed an improved evolutionary method to deploy UAVs forming a virtual antenna array. In addition, UAV deployment and user association are often jointly optimized to improve system performance. Researchers in [10,11] utilized UAVs as aerial base stations to deliver content to users. They employed the K-means algorithm to cluster users, and positioned UAVs at the centroid of each cluster while serving the corresponding user group.

### 1.2. Our Work and Contributions

Most previous studies [5,9,10,11] primarily focused on UAV-enabled wireless communication networks, overlooking the computing requirements of users. Furthermore, few previous studies [5,6,7,8,9,10,11] considered heterogeneous UAV-enabled networks, i.e., deploying UAVs with different communication and computing capabilities. Motivated by these issues, in this paper, we study the UAV deployment and user association problem to save the energy consumption of UAVs in a more extensive heterogeneous multi-UAV-enabled MEC networks. Here, we note that our work is different from [8] in terms of the system model, optimization variables, algorithms, as well as analytical results. Different from the fixed hovering positions of single UAV in [8], multiple heterogeneous UAVs are considered and the deployment of UAVs are jointly optimized with user association, aiming to achieve further system performance improvement. The main contributions of this work are listed as follows.

- Firstly, we consider heterogeneous UAV-enabled MEC networks and formulate a problem to minimize the UAV energy consumption, where UAVs with varying communication and computing capabilities co-exist. The problem is decomposed into two parts, i.e., user association sub-problem and UAV deployment sub-problem, which are solved jointly.
- Secondly, the user association sub-problem is modeled as a semi-discrete optimal transport problem. We prove the existence of the optimal solution by using optimal transport theory (OTT) and characterize the solution space. The dragonfly algorithm (DA) is introduced to find the optimal deployment of multiple UAVs. The fitness of the DA is determined by formulating the optimal association scheme and subsequently computing the corresponding energy consumption.
- Finally, we test the performance of the proposed algorithm with different user distribution models. Compared with the benchmarks, the proposed algorithm that jointly optimizes user association and UAV deployment reduces the energy consumption by up to 39%. Moreover, the convergence and complexity analysis of our algorithms are provided.

## 2. System Model and Problem Formulation

**Figure 1:**
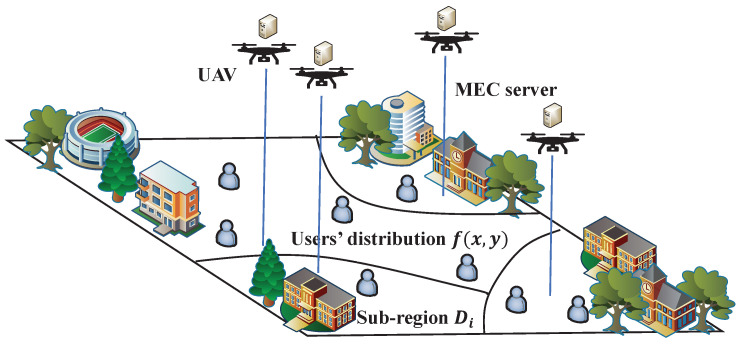
System model.

In our proposed system, as shown in Figure 1, a geographical area $D\subset R^{2}$ is considered, where *N* users are located according to a given distribution f(x,y) over the two-dimensional plane. The network capacity is supported by *K* heterogeneous UAVs, equipped with communication devices and computing servers. As the users’ computation capability is limited, their computing tasks are uploaded to MEC servers equipped on UAVs, which results in *K* disjoint UAV-serving sub-regions. We use the three-dimensional (3D) coordinate $\left(x_{k},y_{k},h_{k}\right),k\in K$ to denote the UAV and $D_{k}$ to denote the corresponding serving region. In each sub-region $D_{k},k\in K$, users first upload their computation tasks to the associated UAV, then wait for the MEC server to calculate these tasks, and finally receive the results.

### 2.1. Computing and Computing Model

In this study, we consider the UAV-enabled networks within urban environments. To achieve this, we utilize the probabilistic channel model presented in [12], which includes both LoS and non-line-of-sight (NLoS) transmission models. In UAV-to-ground communications, the probability of LoS links is dependent on the elevation angle between the user and UAV, as well as the density and height of obstacles. Therefore, we express the path loss between UAV *i* and a user located at (x,y) as

(1) $$\begin{array}{c} PL_{i}^{uav}\left(x,y\right)=K_{o}d_{i}^{2}\left(x,y\right)\left[P_{i}^{\mathrm{LoS}}\mu_{\mathrm{LoS}}+P_{i}^{\mathrm{NLoS}}\mu_{\mathrm{NLoS}}\right], \end{array}$$

where $K_{o}={\left(\frac{4\pi f_{c}}{c}\right)}^{2}$, *c* is the speed of light, $f_{c}$ is the carrier frequency, $d_{i}\left(x,y\right)$ is the distance between the user at (x,y) and UAV *i* expressed as

(2) $$d_{i}\left(x,y\right)=\sqrt{{\left(x-x_{i}\right)}^{2}+{\left(y-y_{i}\right)}^{2}+h_{i}^{2}},$$

where $\mu_{\mathrm{LoS}}$ and $\mu_{\mathrm{NLoS}}$ are the shadow fading random variable for LoS link and NLoS link, respectively. The probability of the LoS and NLoS link can be expressed as

(3) $$P_{i}^{\mathrm{LoS}}=\frac{1}{1+a\exp\left(-b\left(\theta_{i}-a\right)\right)},$$

and

(4) $$P_{i}^{\mathrm{NLoS}}=1-P_{i}^{\mathrm{LoS}},$$

where *a* and *b* denote the environment constants, $\theta_{i}=\sin^{-1}\left(\frac{h_{i}}{d_{i}\left(x,y\right)}\right)$ is the elevation angle. With knowledge of the path loss $PL_{k}\left(x,y\right)$ from the UAV, the rate of a user at (x,y) uploading tasks to a UAV *k* is expressed as

(5) $$R_{k}=\frac{B_{k}}{N_{k}}\log_{2}\left(1+\frac{P_{user}}{PL_{k}\left(x,y\right)\sigma^{2}}\right),$$

where $B_{k}$ is the bandwidth available for UAV *k*, $N_{k}$ is the number of user served by UAV *k*, $\sigma^{2}$ is the power of AWGN, $P_{user}$ is the transmit power of the user. In our analysis, we denote the location of user as e=(x,y), and the location of UAV as $s_{k}$, where k∈K. The task uploading time in sub-region $D_{k}$ can be calculated as [13]

(6) $$t_{c}\left(e,s_{k}\right)={\;\int \int \;}_{D_{k}}\frac{M}{R_{k}}f\left(x,y\right)dxdy,$$

where *M* is the average size of a task. Similar to [14], the computing time for UAV *k* processing tasks in area $D_{k},k\in K$ is calculated as

(7) $$t_{e}\left(e,s_{k}\right)=\frac{{\left(LN_{k}\right)}^{\alpha }}{C_{k}},$$

where *L* is the average computational load for a task, $C_{k}$ is the computing ability of UAV *k*, α is the computing parameter and α>1.

### 2.2. Energy Consumption Model

The energy consumption of UAV primarily includes three main components: communication, computation and hovering. In practical scenarios, the energy consumed by UAV communication is significantly lower than that consumed by the other two [15]. Therefore, this paper does not focus on the energy consumed by UAV communication and instead provides detailed models for the remaining components.

The energy consumption of the UAV for computation at sub-area *k* is expressed as

(8) $$E_{k}^{e}=P_{e}^{k}t_{e}\left(e,s_{k}\right),$$

where $P_{e}^{k}=\kappa {\left(f_{k}\right)}^{3}$, κ is the effective capacitance coefficient [15], and $f_{k}$ is the central processing unit (CPU) frequency of MEC server on UAV *k*.

While the UAV hovers over sub-region $D_{k}$, the duration it spends is mainly communication time and computation time. Communication time is associated with users from sub-region $D_{k}$ offloading their tasks to the UAV. Computation time, on the other hand, represents the duration the UAV takes to process these offloaded tasks. Similar to [16], the minimum power required for *n* rotors of diameter *d* to hover can be expressed as

(9) $$P_{h}=\frac{T^{3/2}}{\sqrt{\frac{1}{2}\pi nd^{2}\rho }},$$

with ρ denoting air density, T=mg denoting the gravity of UAV, *g* being the gravitational constant. Thus, the energy consumption of the UAV for hovering at sub-area *k* is expressed as

(10) $$E_{k}^{h}=P_{h}\left(t_{c}\left(e,s_{k}\right)+t_{e}\left(e,s_{k}\right)\right).$$

### 2.3. Problem Formulation

In the UAV-aided MEC networks, we try to minimize the energy consumption by optimizing the user association and the UAV depolyment. The total energy consumed by the UAV while serving all user devices in the whole region *D* is dependent on the set of partitioned sub-areas $D=\left\{D_{k},k\in K\right\}$ and the UAV deployment location $L=\left\{l_{k},k\in K_{u}\right\}$, which can be formulated as

(11) $$E\left(D,L\right)=\sum_{k=1}^{K}\left(E_{k}^{e}+E_{k}^{h}\right).$$

The optimization problem can be formulated as

(12a)P1:minD,LE(D,L),(12b)s.t.Dm∩Dn=⌀∀m,n∈K,(12c)⋃k∈KDk=D,(12d)xumin≤xk≤xumax,1∀k,(12e)yumin≤yk≤yumax,1∀k,(12f)humin≤hk≤humax,1∀k,

where (12b) and (12c) are the constrains that the sub-regions do not overlap and cover the entire area, and $\left[x_{u}^{\min},x_{u}^{\max}\right]\times \left[y_{u}^{\min},y_{u}^{\max}\right]\times \left[h_{u}^{\min},h_{u}^{\max}\right]$ restricts the horizontal location of the UAVs in (12d), (12e) and (12f).

## 3. Problem Analysis and Algorithm Design

Problem P1 is an NP-hard problem, with the coupled region partition variable D and UAV deployment L. To solve this problem, we partition problem P1 into two subproblems, namely user association optimization and UAV deployment optimization. The procedure is described below.

### 3.1. Optimization of User Association

The region partitions $D_{k},\forall k\in K$ are continuous and coupled in the sub-problem P2. To solve this sub-problem, we first prove the optimal solution exists via introducing optimal transport theory [17], and then characterize the optimal solution. At last, we propose a low-complexity iterative algorithm to approach the optimal region partitioning. The user association sub-problem can be rewritten as

(13a)P2:minD  E(D,L^),(13b)s.t.(12b)(12c).

As users follow a continuous distribution f(x,y), and UAVs can be regarded as discrete points, sub-problem P2 with fixed UAV location L can be seen as a semi-discrete optimal transport problem. Thus, sub-problem P2 is equivalent to matching users to the UAVs with the minimum energy consumption.

**Theorem** **1.** *Problem P2 has an optimal solution.*

**Proof.**  Let $d_{k}=\int_{D_{k}}f\left(x,y\right)dxdy$, for ∀k∈K, $F\left(e,s_{k}\right)=\frac{M}{\log_{2}\left(1+\frac{P_{user}}{PL_{k}\left(x,y\right)\sigma^{2}}\right)}$ and $c\left(e,s_{k}\right)=P_{h}F\left(e,s_{k}\right)$. For any given $s_{k}$, $c(e,s_{k})$ is continuous. We have $\underset{e\to e_{0}}{lim inf}c\left(e,s_{k}\right)\geq c\left(e_{0},s_{k}\right)$, so $c(e,s_{k})$ is lower semi-continuous. Then, Lemma 1 is used from optimal transport theory [18]:

**Lemma** **1.** *Consider continuous probability measure f and discrete probability measure λ in *Ω*. Let L:Ω→Ω be a transport map from f to λ and C(x,E(x)):Ω×Ω→[0,∞) be the cost function of L. Then, for any semi-continuous cost function, the optimal transport map from f to λ exists, which minimizes the total transport cost $\int_{\Omega }C\left(x,E\left(x\right)\right)f\left(x\right)dx$.*

According to Lemma 1, the sub-problem P2 has an optimal solution.    □

**Theorem** **2.** *To achieve minimum energy consumption in the UAV aided MEC networks, the optimal region partition is given by*

(14) $$\begin{array}{c} D_{k}^{*}=\left\{\left(x,y\right):P_{h}\frac{N_{k}}{B_{k}}F\left(e,s_{k}\right)+\frac{\alpha L^{\alpha }N_{k}^{\alpha -1}}{C_{k}}\left(P_{h}+P_{e}\right)\leq P_{h}\frac{N_{n}}{B_{n}}F\left(e,s_{n}\right)+\frac{\alpha L^{\alpha }N_{k}^{\alpha -1}}{C_{n}}\left(P_{h}+P_{e}\right),\forall n\neq k\in K\right\}, \end{array}$$

**Proof.**  According to Theorem 1, optimal region partitions $D_{k}^{*},k\in K$ exist, which are the solutions to problem (13). Now, we consider another region partition scheme ${\tilde{D}}_{k},k\in K$ as an example. Taking a coordinate $z_{0}=\left(x_{0},y_{0}\right)\in D_{m}$ and a circle area $B_{\tau }$ with the center $z_{0}$ and radius τ>0, the region partition ${\tilde{D}}_{k},k\in K$ is generated from the optimal partition as

(15) $$\left\{\begin{array}{c} {\tilde{D}}_{m}=D_{m}\setminus B_{\tau }\left(v_{0}\right),\\ {\tilde{D}}_{n}=D_{n}\cup B_{\tau }\left(v_{0}\right),\\ {\tilde{D}}_{k}=D_{k},\;k\neq m,n. \end{array}\right.$$

We denote $d_{\tau }={\;\int \int \;}_{B_{\tau }}f\left(x,y\right)dxdy$ and ${\tilde{d}}_{k}={\;\int \int \;}_{{\tilde{D}}_{k}}f\left(x,y\right)dxdy$. As the region partition $D_{k}^{*},k\in K$ is optimal, a better solution cannot be achieved by any variation of the optimal partitions ${\tilde{D}}_{k},k\in K$. We have

(16) $$\begin{array}{c} \sum_{k=1}^{K}\int_{D_{k}}\left(E_{k}^{p}+E_{k}^{h}\right)\leq \sum_{k=1}^{K}\int_{{\tilde{D}}_{k}}\left(E_{k}^{p}+E_{k}^{h}\right). \end{array}$$

Now, we subtract the common items on both sides of the equation, yielding

(17) $$\begin{array}{c} \int_{D_{m}}\left(E_{m}^{p}+E_{m}^{h}\right)+\int_{D_{n}}\left(E_{n}^{p}+E_{n}^{h}\right)\leq \int_{D_{m}\setminus B_{\tau }\left(v_{0}\right)}\left(E_{m}^{p}+E_{m}^{h}\right)+\int_{D_{n}\cup B_{\tau }\left(v_{0}\right)}\left(E_{n}^{p}+E_{n}^{h}\right). \end{array}$$

We denote $h\left(d_{k}\right)=\frac{{\left(Ld_{k}\right)}^{\alpha }}{C_{k}}$; (17) can be simplified as

(18) $$\begin{array}{cc} {\;\int \int \;}_{B_{\tau }}\frac{P_{h}F\left(e,s_{k}\right)}{B_{k}} & dxdy+\left(P_{h}+P_{e}^{k}\right)\left(h\left(d_{m}\right)-h\left(d_{m}-d_{\tau }\right)\right)\\ \; & \leq {\;\int \int \;}_{B_{\tau }}\frac{P_{h}F\left(e,s_{k}\right)}{B_{k}}dxdy+\left(P_{h}+P_{e}^{k}\right)\left(h\left(d_{n}\right)-h\left(d_{n}-d_{\tau }\right)\right). \end{array}$$

We divide both sides of the inequality by $d_{\tau }$ and take the limit when τ→0. We have

(19) $$\begin{array}{c} D_{k}^{*}=\left\{\left(x,y\right):P_{h}\frac{N_{k}}{B_{k}}F\left(e,s_{k}\right)+\left(P_{h}+P_{e}^{k}\right)h^{\prime }\left(d_{m}\right)\leq P_{h}\frac{N_{n}}{B_{n}}F\left(e,s_{n}\right)+\left(P_{h}+P_{e}^{k}\right)h^{\prime }\left(d_{n}\right),\forall n\neq k\in K\right\}, \end{array}$$

and (19) shows that we assign a user at $\left(x_{0},y_{0}\right)$ to a cell. Consequently, a tractable expression of the optimal region partition is given in Theorem 2.    □

An explicit characterization for the optimal region partition is easily given, but this expression is not practical. Therefore, Algorithm 1 is proposed to approximate the optimal region partition, the main idea of which is to introduce an damping argument γ to make the algorithm converge. Specifically, *Z* is the maximum number of iterations, continuous variable $\phi_{i}^{t}\left(x,y\right)\in \left[0,1\right]$ indicates whether the user at (x,y) is served by the UAV *i*. For example, $\phi_{i}^{t}\left(x,y\right)=1$ represents the fact that user at (x,y) belongs to sub-region $D_{i}$, while $\phi_{i}^{t}\left(x,y\right)=0$ means the opposite. At iteration *t*, if user at (x,y) associates with UAV *i*, then $\phi_{i}^{\left(t+1\right)}\left(x,y\right)=1-\gamma \left(1-\phi_{i}^{t}\left(x,y\right)\right)$; otherwise, $\phi_{i}^{\left(t+1\right)}\left(x,y\right)=\gamma \phi_{i}^{t}\left(x,y\right)$. After that, the percentage of users in sub-region $D_{i}$ is calculated by $d_{i}=\int_{D}\phi_{i}^{\left(t+1\right)}\left(x,y\right)f\left(x,y\right)dxdy,\forall i\in K$ and assigns each user according to (14). With the increase in *t*, $\phi_{i}$ converges, and region partition approaches the optimal region partition.
**Algorithm 1** Iterative Algorithm for User Association**Input:** Total number of users *N* and distribution function f(x,y), the UAV location set L.**Output:** The optimal region partition $D_{i}^{t},\forall i\in K$.
1:Let t=1, initialize user association Ω and let $\phi_{i}^{t}\left(x,y\right)=0,\forall i\in K$.2:**while** *t*≤ Z **do**3:   γ←1−1/t4:   Compute $\phi_{i}^{t+1}\left(x,y\right)$$=\left\{\begin{array}{cc} 1-\gamma \left(1-\phi_{i}^{t}\left(x,y\right)\right), & \;\mathrm{if}\;\left(x,y\right)\in D_{i}^{t}.\\ \gamma \phi_{i}^{t}\left(x,y\right), & \;\mathrm{otherwise}.\; \end{array}\right.$5:   Compute $d_{i}=\int_{D}\phi_{i}^{\left(t+1\right)}\left(x,y\right)f\left(x,y\right)dxdy,\forall i\in K$6:   t←t+17:   Update user association using (14).8:**end while**9:$D_{i}^{*}\leftarrow D_{i}^{t},\forall i\in K$.

### 3.2. Optimization of UAV Deployment

The sub-problem of deployment optimization for UAVs is given as P3. Problem P3 is an NP-hard problem. As computing gradients of *L* in $E(\hat{D},L)$ is computationally difficult, using some gradient-based methods, e.g., convex optimization, alternating directional method of multipliers (ADMM), successive convex approximation (SCA), etc., is not suitable for this situation. Evolutionary algorithms, as a representative of non-gradient optimization, have received widespread attention. Evolutionary algorithms study the complex collective behavior of systems composed of multiple simple agents that can interact with other agents locally and with their surrounding environment. Among these algorithms, the dragonfly algorithm has shown superiority and outperformance compared to other evolutionary algorithms [19]. Therefore, we use the DA to solve the sub-problem P3.

(20a)P3:minLE(D^,L),(20b)s.t.(12d)(12e)(12f).

In Algorithm 2, our proposed algorithm operates with a swarm of dragonflies, where each dragonfly represents a possible solution $X_{i}$, i.e., a UAV deployment scheme. These solutions are updated iteratively to find the best scheme according to a given fitness function, which quantifies how well a potential solution performs with respect to the specific aim. For the purpose of UAV location optimization, the fitness function is set as the energy consumption E(D,L). The positions and velocities of the dragonflies are updated by separation, alignment, cohesion, food attraction, and enemy avoidance, as well as migration behavior. The position update of the dragonfly *i* in the iteration *t* is given by

(21) $$X_{i,t+1}=X_{i,t}+V_{i,t+1},$$

where $X_{i,t}$ is the position of the dragonfly *i* in the iteration *t* and $V_{i,t+1}$ is the velocity update for the iteration t+1. The velocity of each dragonfly is updated according to the following formula:

(22) $$V_{i,t+1}=w\times V_{i,t}+S_{i}+A_{i}+C_{i}+F_{i}+E_{i}+M_{i},$$

where term $w*V_{i,t}$ captures the dragonfly’s inertia, reflecting its tendency to persist in its current motion. $S_{i},A_{i}$, and $C_{i}$ correspond to the dragonfly’s separation, alignment, and cohesion behaviors, respectively. Within a neighborhood radius *r*, the dragonfly maintains distance from neighbors (separation), aligns its direction with the average heading of neighbors (alignment), and is pulled towards the average position of neighbors (cohesion). The remaining terms, $F_{i},E_{i}$, and $M_{i}$, guide the dragonfly towards food sources, away from threats, and towards migration points, representing food attraction, enemy avoidance, and migration behaviors, respectively [19].

In order to enhance the randomness, unpredictability, and exploration capability of the dragonflies, they are programmed to search space via a random walk (known as Levy flight) in the absence of neighboring solutions. In this case, the position of the dragonflies is updated using the following equation:

(23) $$X_{t+1}=X_{t}+\mathrm{Levy}\;\times \;X_{t},$$

where Levy is determined by the dimension of the position vectors given in [19]. The joint user association and UAV deployment optimization is shown in Algorithm 2.
**Algorithm 2** Joint User association and Deployment Optimization for Energy-efficient UAV-aided MEC Networks**Input:** Population size, termination condition *z* and distribution function f(x,y).**Output:** UAV location $L^{*}=Gbest$, the optimal region partition $D^{*}$ with $L^{*}$.1:Initialize population of dragonflies2:**for** each dragonfly *i* **do**3:   Initialize position $X_{i}$ with a random vector.4:   Initialize velocity $V_{i}$ with a random vector.5:   Evaluate $X_{i}$ by $fitness\left(X_{i}^{0}\right)=E\left(\hat{D},X_{i}^{0}\right),\forall i$.6:**end for**7:**while** *t* ≤ Z **do**8:   **for** each dragonfly *i* **do**9:     Optimize user association D with fixed UAV location $X_{i}^{t}$ by **Algorithm** 1.10:     Calculate fitness of all dragonflies $fitness\left(X_{i}^{t}\right)=E\left(\hat{D},X_{i}^{t}\right),\forall i$.11:     Calculate $S_{i}^{t}$, $A_{i}^{t}$, $C_{i}^{t}$, $F_{i}^{t}$, $E_{i}^{t}$, and $M_{i}^{t}$ according to [19].12:     **if** a dragonfly has at least one neighbouring dragonfly **then**13:        Update velocity $V_{i}^{t}$ according to (22).14:        Update position $X_{i}^{t}$ according to (21).15:     **else**16:        Update velocity $V_{i}^{t}$ according to (23).17:     **end if**18:   **end for**19:**end while**20:Return the best solution Gbest

## 4. Numerical Results

In our simulations, we assume that four UAVs with different capabilities are deployed to serve users in a region of size 1 km × 1 km, where two UAVs are Low-capability UAVs (sUAVs), one UAV is Medium-capability UAV (mUAV) and one UAV is Large-capability UAV (lUAV). The transmit power of UAVs is 10 W, the bandwidth of sUAV, mUAV and lUAV is 5  MHz, 8  MHz, and 10  MHz, the CPU frequency of MEC servers on sUAV, mUAV and lUAV is 5  GHz, 8  GHz, and 10  GHz, respectively. Also, the computation capacities of sUAV, mUAV and lUAV are 5000 GFLOPs/s, 8000 GFLOPs/s and 10,000 GFLOPs/s, respectively [20]. Other simulation parameters are listed in Table 1.

**Figure 2:**
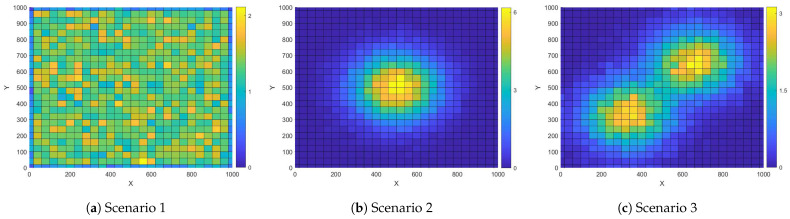
User distribution with (**a**) uniform distribution, (**b**) unimodal distribution, (**c**) bimodal distribution.

In all simulations, three classical user distributions are considered: uniform, unimodal [21], and bimodal [22]. We first offer the probability density function for uniform distrition as

(24) $$f\left(x,y\right)=\frac{N}{\left|D\right|},$$

where D represents the total area of the UAV-serving network. The uni-modal user probability density function is characterized using a two-dimensional truncated Gaussian distribution [21] as

(25) $$f\left(x,y\right)=\frac{N}{\eta }\exp\left[-{\left(\frac{x-\mu_{x}}{\sqrt{2}\sigma_{x}}\right)}^{2}\right]\exp\left[-{\left(\frac{y-\mu_{y}}{\sqrt{2}\sigma_{y}}\right)}^{2}\right],$$

where $\eta =2\pi \sigma_{x}\sigma_{y}\mathrm{erf}\left(\frac{L_{x}-\mu_{x}}{\sqrt{2}\sigma_{x}}\right)\mathrm{erf}\left(\frac{L_{y}-\mu_{y}}{\sqrt{2}\sigma_{y}}\right)$, $\mu_{x},\sigma_{x},\mu_{y},\;$and $\sigma_{y}$ are the mean and standard deviation values of *x* and *y* coordinates, and $\mathrm{erf}\left(z\right)=\frac{2}{\sqrt{\pi }}\int_{0}^{z}e^{-t^{2}}\;dt$. $\left(\mu_{x},\mu_{y}\right)$ represents the center of the hotspot, and the density of the users around the center is inversely proportional to the values $\sigma_{x}$ and $\sigma_{y}$. Similarly, the bi-modal user distribution can be viewed as a combination of two truncated Gaussian distributions [22], with its probability density function expressed as

(26) $$f\left(x,y\right)=\lambda f_{1}\left(x,y\right)+\left(1-\lambda \right)f_{2}\left(x,y\right),$$

where 0≤λ≤1 represents a weight factor. $f_{1}\left(x,y\right)$ and $f_{2}\left(x,y\right)$ represent two truncated Gaussian distribution models. In our bimodal user distribution, we set mean values as $\mu_{x_{1}}=\mu_{y_{1}}=330$, $\mu_{x_{2}}=\mu_{y_{2}}=660$; the variance values are $\sigma^{2}=\sigma_{x_{1}}^{2}=\sigma_{y_{1}}^{2}=\sigma_{x_{2}}^{2}=\sigma_{y_{2}}^{2}=20,000$, and λ=0.5. These three models correspond to user uniformly distributed scenario, single hot spot scenario and multiple hot spots scenarios such as carnival in a park or sports events. The user distributions are shown in Figure 2.

**Figure 3:**
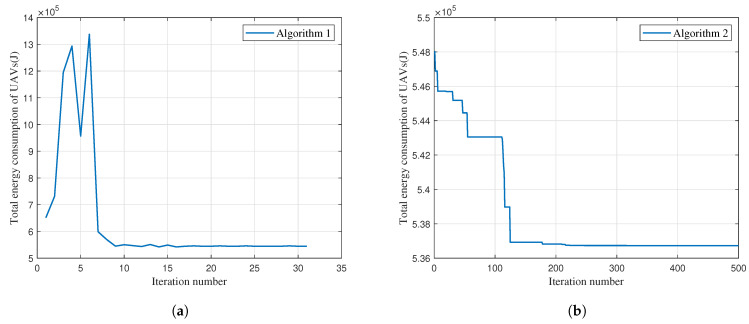
Convergence speed of Algorithms 1 and 2. (**a**) Algorithm 1 convergence speed. (**b**) Algorithm 2 convergence speed.

Figure 3 shows the convergence performance of Algorithms 1 and 2. It can be observed that the total energy consumption of the UAVs is $6.5\times 10^{5}$ J after the initial iteration. As the number of iterations progresses, the UAV energy consumption first increases and then starts to decline. By the 10th iteration, the consumption stabilizes, settling at approximately $5.5\times 10^{5}$ J. Also, Algorithm 2 converges within 200 iterations. The computational complexities for each iteration in Algorithms 1 and 2 are given by $O\left(KN\right)$ and $O\left(SK^{2}N\right)$[23], where *S* represents the number of dragonflies. Then, the proposed Algorithms 1 and 2 is compared with the benchmarks as follows (SNR is short for signal-to-noise ratio):

• Uniform+OTT: UAVs are deployed uniformly and users access the BS by the OTT algorithm.
• Uniform+SNR: UAVs are deployed uniformly and users access the BS with the largest SNR.
• K-means: The UAV deployment and user association are determined by the K-means algorithm [11].

### 4.1. Superiority of the Proposed OTT-Based User Association Algorithm

**Figure 4:**
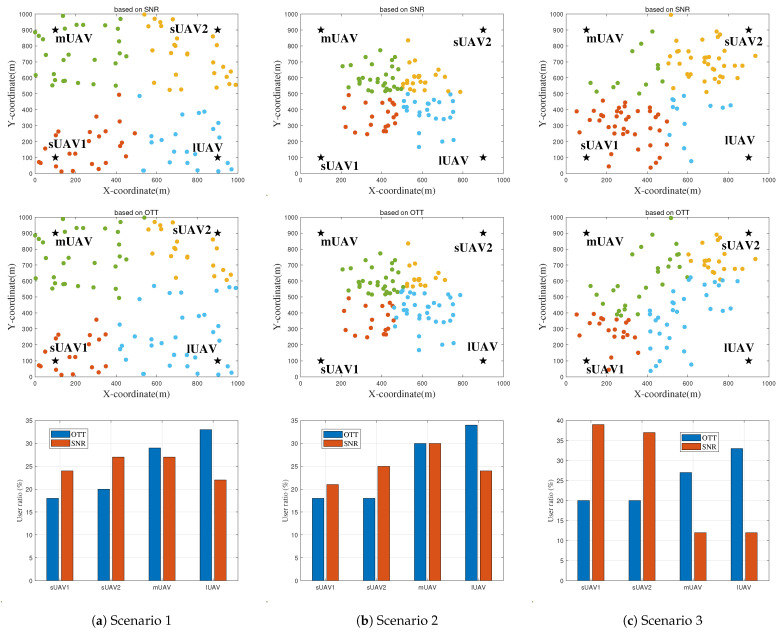
User association with (**a**) uniform distribution, (**b**) unimodal distribution, (**c**) bimodal distribution. UAVs are represented by black stars, users are represented by dots, and different colored dots represent users belonging to different UAV serving regions.

**Figure 5:**
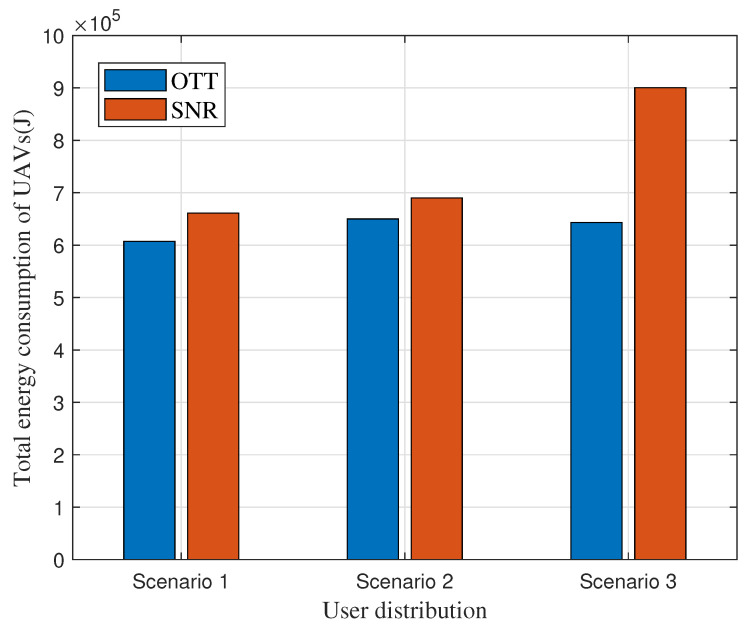
Comparison of total energy in different scenarios.

This subsection investigates the proposed OTT-based user association algorithm, focusing on its corresponding energy consumption across three distinct scenarios. To highlight the advantages of this approach, the classical SNR-based association scheme, a prevalent method in wireless networks, is used as a benchmark. The SNR method follows the maximum signal-to-noise rule to associate users with UAVs. Furthermore, in all the considered scenarios, UAVs are uniformly deployed in the target area. The setup includes two sUAVs located at coordinates (100, 100) and (100, 900), a mUAV at (900, 100), and a lUAV at (900, 900). The outcome of these evaluations is visualized in Figure 4 and Figure 5, illustrating the performance and benefits of the proposed OTT-based user association algorithm.

In Figure 4a corresponding to Scenario 1, we observe that the target region is partitioned into four sectors. Each of these cell boundaries is primarily determined by the signal power strength received from different UAVs. This approach, however, fails to leverage the significant difference in bandwidth and computational resources across the varying UAVs. Illustrated in the middle part of Figure 4a, the OTT-based scheme adjusts the cell boundaries of each UAV, taking into account their distinct capabilities. Consequently, under the OTT-based association, the proportion of users served by $sUAV_{1}$ and $sUAV_{2}$ reduces from 24% and 27% to 17.5% and 20%, respectively. Concurrently, the fraction of users within the coverage area of lUAV increases from approximately 22% to 33%. This shows how the OTT-based scheme optimizes user association by effectively leveraging the unique capabilities of each UAV.

In Scenario 2 depicted in the top of Figure 4b, only approximately 24% of users fall within the coverage area of the lUAV. This uneven distribution results in unbalanced communication and computing loads. In contrast, as shown in the middle of Figure 4b, the proposed OTT-based association scheme manages more balanced loads. This balance arises from its capacity to perceive and adapt to the user distribution. Particularly, the regions covered by the lUAV expand to shoulder more communication and computing loads. More specifically, the proportion of ground users served by the lUAV increase from 24% to 34%. Simultaneously, the user coverage by $sUAV_{1}$ and $sUAV_{2}$ decreases from about 21% to 17.5%, and from 25% to 17.5%, respectively. This representation of the OTT-based scheme further demonstrates its ability to efficiently distribute network resources and maintain balanced loads across various UAVs.

In Scenario 3, we note that a significant proportion of users (about 75%) is associated with the small UAVs (sUAVs) under the SNR-based scheme from the top of Figure 4c. However, when the OTT-based association scheme is employed, the medium (mUAV) and large UAVs (lUAV) extend their coverage areas to alleviate the computing pressure on the sUAVs located near the hotspots. Specifically, the proportion of users served by the mUAV and lUAV rises from 12.5% to 27%, and from 12.5% to 33%, respectively. Concurrently, the number of users covered by $sUAV_{1}$ and $sUAV_{2}$ decreases from around 39% to 20%, and from 36% to 20%, respectively. These changes occur because the OTT-based association scheme considers not only the signal strength received by users but also the user distribution and UAV location. The results drawn from this scenario affirm that the proposed OTT scheme can significantly balance the load among UAVs with diverse capabilities, offering a marked improvement over the baseline scheme.

The energy consumption simulations also verify our analysis about the SNR-based scheme and the proposed OTT-based association scheme shown in Figure 5. As discussed above, due to the comprehensive consideration of bandwidth, power, and communication resources, the OTT-based association demonstrated lower energy consumption in all scenarios. In Scenario 1, the energy consumption for OTT-based association is $6\times 10^{5}\;$J versus $6.6\times 10^{5}\;$J for SNR; in Scenario 2, it is with $6.5\times 10^{5}\;$J for OTT and $6.95\times 10^{5}\;$J for SNR. The most substantial difference occurred in Scenario 3, where the OTT approach consumes $6.4\times 10^{5}\;$J, while the SNR approach consumes $9\times 10^{5}\;$J. Specifically, there was a decrease of approximately 10% in Scenario 1, 6.5% in Scenario 2, and an impressive 28.9% reduction in Scenario 3 compared to the SNR-based method. These quantifiable results highlight the OTT-based user association algorithm as more energy efficient for UAV-enabled MEC networks, confirming its advantages over the traditional SNR-based approach.

### 4.2. Superiority of the Proposed Joint User Association and UAV Deployment Optimization Algorithm

**Figure 6:**
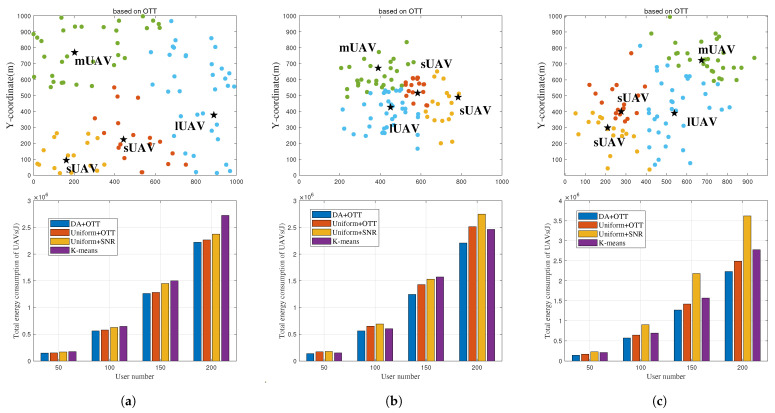
User association, UAV deployment and the energy consumption of Algorithm 2 versus different scenarios. (**a**) Scenario 1. (**b**) Scenario 2. (**c**) Scenario 3. UAVs are represented by black stars, users are represented by dots, and different colored dots represent users belonging to different UAV serving regions.

Figure 6 shows the superiority of Algorithm 2 which jointly optimizes user association and UAV deployment. In Scenario 1, sUAV1, sUAV2, sUAV1 and lUAV are placed at (443.7, 224.3), (161.4, 94.3), (202.6, 770.4) and (888.5, 377.2), respectively, which scatter on the target region as users are uniformly distributed. The proposed algorithm outperforms the ’Uniform + OTT’, ’Uniform + SNR’ and ’K-means’ benchmarks by about 3%, 6.5% and 13.1%, respectively. In Scenario 2, sUAV1, sUAV2, sUAV1 and lUAV are placed at (584, 515.4), (783.2, 489.2), (389.4, 672.3) and (450.3, 426.5), respectively, which converge to the the hotspot area. The proposed Algorithm outperforms the ’Uniform+OTT’, ’Uniform+SNR’ and ’K-means’ benchmarks by about 12.1,%, 20% and 11.1%, respectively. In Scenario 3, sUAV1, sUAV2, sUAV1 and lUAV are placed at (277, 401.4), (210.2, 299.5), (672.3, 722.8) and (539,391), respectively, which are optimized to approach the two hotspot areas. The proposed algorithm outperforms the ’Uniform + OTT’, ’Uniform + SNR’ and ’K-means’ benchmarks by about 10.3,%, 38.31% and 26.4%, respectively. In addition, compared to the uniform + SNR scheme, optimizing user association with the OTT-based algorithm can reduce energy consumption by 31.3%, and the proposed Algorithm 2 can reduce energy consumption by 39%. The results indicate that the system performance can be improved by individually using OTT-based user association algorithm, with the given UAV deployment location. For further enhancement of system performance, it is crucial to jointly optimize both the association of users and the deployment of UAVs. In all scenarios, the overall energy consumption of UAVs increases as the number of users grows.

## 5. Conclusions

In this paper, we investigated the energy consumption minimization problem in heterogeneous UAV-aided MEC networks, where UAVs have different communication and computing capabilities. In particular, we proposed an algorithm to jointly optimize user association and UAV deployment. In doing so, the DA algorithm was adopted to find the deployment of multiple UAVs. The fitness in DA was determined by formulating the optimal association scheme and subsequently computing the corresponding energy consumption. The existence and characteristics of the optimal user association were obtained by using optimal transport theory, and an iteration algorithm was developed to approach the optimal user association. Numerical results showed that the proposed algorithm can reduce the energy consumption by up to 39%.

## Figures and Tables

**Table 1:** Simulation parameters.

Parameters	Description	Value
$f_{c}$	Carrier frequency for UAVs	2 GHz
*c*	Speed of light	$3\times 10^{8}$ m/s
*m*	Weight of UAVs	50 kg
*N*	Total number of users	100
*M*	Data size of each task	1 Mb
κ	Effective capacitance coefficient	$10^{-28}$
α	Computing parameter	1.2
ρ	Air density	$1.29\;\mathrm{Kg}/m^{3}$
*n*	Rotor numbers of UAVs	4
*d*	Rotor diameter	1 m
*L*	Average computational load for a task	60 GFLOPs
$P_{user}$	User transmit power	2 W
$\sigma^{2}$	Noise power	−110 dBm
$\mu_{\mathrm{LoS}}$	Additional path loss for LoS	3 dB
$\mu_{\mathrm{NLoS}}$	Additional path loss for NLoS	23 dB
*a*	The LoS probability constant	8.96
*b*	The LoS probability constant	0.04

## Data Availability

Not applicable.
